# Studying health-related internet and mobile device use using web logs and smartphone records

**DOI:** 10.1371/journal.pone.0234663

**Published:** 2020-06-12

**Authors:** Ruben L. Bach, Alexander Wenz

**Affiliations:** 1 Collaborative Research Center 884 Political Economy of Reforms, University of Mannheim, Mannheim, Germany; 2 Institute for Social and Economic Research, University of Essex, Colchester, United Kingdom; Universiteit Twente, NETHERLANDS

## Abstract

Many people use the internet to seek information that will help them understand their body and their health. Motivations for such behaviors are numerous. For example, users may wish to figure out a medical condition by searching for symptoms they experience. Similarly, they may seek more information on how to treat conditions they have been diagnosed with or seek resources on how to live a healthy life. With the ubiquitous availability of the internet, searching and finding relevant information is easier than ever before and a widespread phenomenon. To understand how people use the internet for health-related information, we use data from a sample of 1,959 internet users. A unique combination of data containing four months of users’ browsing histories and mobile application use on computers and mobile devices allows us to study which health websites they visited, what information they searched for and which health applications they used. Survey data inform us about users’ socio-demographic background, medical conditions and other health-related behaviors. Results show that women, young users, users with a university education and nonsmokers are most likely to use the internet and mobile applications for health-related purposes. On search engines, internet users most frequently search for pharmacies, symptoms of medical conditions and pain. Moreover, users seem most interested in information on how to live a healthy life, alternative medicine, mental health and women’s health. With this study, we extend the field’s understanding of who seeks and consumes health information online, what users look for as well as how individuals use mobile applications to monitor their health. Moreover, we contribute to methodological research by exploring new sources of data for understanding humans, their preferences and behaviors.

## Introduction

Many people use the internet to seek information that will help them understand their body and their health [1–3]. Motivations for such behaviors are numerous. For example, users may wish to figure out a medical condition by searching for symptoms they experience. Similarly, they may seek more information on how to treat conditions they have been diagnosed with or seek resources on how to live a healthy life. With the ubiquitous availability of the internet, searching and finding relevant information is easier than ever before and a widespread phenomenon. In the U.S., up to two out of three adults regularly search for health information online [4, 5]. Likewise, two out of five internet users in Germany search the internet for health information before their doctor’s appointment, and around half of internet users after their appointment [6].

In addition, the rise of health applications (apps) on mobile devices such as smartphones and tablets, as well as accompanying health and fitness trackers (“wearables”), make it possible for people to track their health and fitness without the help of medical professionals and at lower costs. Just like researching health information online, the use of health apps on mobile devices is spreading rapidly. For example, about one in five smartphone owners in the U.S. used a health app in 2012 [7]. Likewise, about one in four U.S. citizens regularly or occasionally use health apps for self-diagnosis [8] and up to 45% of U.S. citizens report using a mobile phone or tablet to manage their health [5]. The numbers for health app use in Germany are very similar: Around two out of three smartphone owners used a health app in 2019 [6]. Moreover, the number of health apps in Apple’s App Store is estimated to be about 90,000 [9].

Understanding who uses health apps on mobile devices and searches and consumes what kind of health information on the internet is crucial for several reasons. Health apps and mobile devices are used to improve self-reflection [10], change behaviors [11] and track physical activity [12]. Likewise, health information collected from the internet influences users’ decisions about their own health and decisions they make for others (e.g., their children) [13]. Similarly, it may affect users’ decisions when to go see a physician or to change eating habits and physical activity [14–16]. Moreover, health-related internet use has the potential to reduce shortcomings in health knowledge for certain subgroups of the population, such as individuals with lower education [17]. Online health information may also influence how users treat conditions or symptoms they experience. However, (mis)information disseminated via the internet may also drive and facilitate the emergence of phenomena such as the spread of anti-vaccination sentiments [18]. This point is exacerbated by the fact that users often do not check the source and data of health information found online [19].

In this paper, we study health-related internet and app use by relying on a unique data set covering passively tracked browsing behavior of 1,959 German internet users over a period of four months in 2017. For some of these users, browsing behavior as well as app use was in addition monitored on smartphones and tablets during the same time period. The data were collected by a commercial vendor who keeps a pool of participants who are occasionally invited to answer short surveys for money.

Previous work mostly relied on self-reported measures of online behavior through questionnaires, which is often inaccurate due to recall error, and previous research often used samples small in size. Our approach of passively tracking browsing behavior addresses these limitations and allows more accurate and detailed insights into how people use the internet. In addition to the tracking data, participants also provided socio-demographic information, information on health issues and information on other lifestyle behaviors that may influence their health (e.g., exercising and smoking). This combination of web logs and survey data creates a unique data source for studying individuals’ health activities in the online world, going far beyond previous research. Overall, we demonstrate what the field can learn from such records of individuals’ online and app activities and point to avenues for future research.

Using these data, we examine who engages in health-related internet and app use and to what extent. That is, we study how online health information searches and app use are associated with socio-demographic characteristics, health conditions and health-related lifestyle behaviors. Results indicate that women, young users and users who have a university degree are more likely to engage in health-related internet/app use. We find limited evidence that health-related internet/app use is related to health conditions, but that it is related with lifestyle behaviors such as smoking. Overall, however, frequent use of health-related apps is not a widespread phenomenon: Only 16% of all app users frequently use a health app. Regarding topics that users engage in the most, we classify participants’ visits to health-related internet domains and apps into broader categories. Our results indicate that users are particularly interested in exercising and weight loss as well as nutrition and alternative medicine. Analysis of health-related search queries made to search engines reveals that users are most interested in finding pharmacies, symptoms of medical conditions, various forms of pain and remedies for health problems.

## Related work

In this section, we review two streams of previous work that are relevant for our study: Correlates of health-related internet and app use and health information search through search engines.

### Correlates of health-related internet and app use

Internet use for health information seeking is associated with a variety of socio-demographic characteristics. Consistent findings are reported regarding education (users with lower education are less likely to seek health information online) [4, 19–21, 21–24] and gender (women more likely) [4, 17, 19, 20, 21, 21, 22, 25]. Moreover, previous research agrees regarding the role of age in determining health-related internet use (younger users more likely) [4, 19, 21, 23, 24]. Some studies also report effects regarding income (users with higher income more likely) [4, 21, 22] and race/ethnicity (non-white users less likely) [4, 25].

Less is known regarding associations between users’ health behavior and health-related internet use. Several studies report that users with “fair” or “poor” health are more likely to use the internet for health purposes [22, 26]. Users who already have a chronic health condition are more likely to seek information online [27], while those who are at risk of getting a medical condition (for example, cancer) rely more often on information obtained from health professionals [23]. Furthermore, there seems to be a positive association between obesity and health-related internet use [28].

Regarding determinants of health-related app use, socio-demographic relationships similar to those mentioned above are reported (e.g., age, education, gender, income) [7, 29–32]. In addition, having a history of chronic medical conditions [33], being obese [31] and engaging in physical activity are all positively associated with health-related app use [30, 33]. Overall, however, regular use of health apps and other digital health solutions is not widespread, even among users with a high digital affinity (see, for example, [34]).

To sum up, previous work identified several socio-demographic correlates of health-related internet and app use, such as age, gender and education. Moreover, a few studies find relationships between users’ health and their online and app activities. One major drawback of all of the studies mentioned here, however, is that they rely on survey data. That is, users self-report whether they engage in health-related internet or app use. However, it is well known that survey reports are often inaccurate as users tend to forget or overestimate actual internet use (see, e.g., [35]). Analyzing web logs and records of mobile device use such as those used in our study offers a more complete and fine-grained picture of users’ online and app activities. Moreover, they allow us to study not only who engages in health-related internet and app use, but also how users obtain their information.

### Health information search through search engines

Besides literature regarding the question of who uses the internet and apps for health-related purposes, previous work studying health-related search queries is relevant for our study. Most of the studies in this domain rely on the analysis of search query data.

Cartright et al. identify health-related search queries (about 20% out of all queries) made to three major search engines in the U.S. over a period of six months [36]. The resulting queries are classified into different foci (symptom, cause or remedy). In addition, the authors train a classifier that predicts what the next focus of a user in a single session will be. Using similar data, White et al. show that users who start a session searching for simple symptoms easily end up searching for serious diseases [13]. Thereby, the authors show that likely innocuous health searches can quickly lead users to seek information about serious, but rare disease with similar symptoms. Using Google search queries, Ginsberg et al. demonstrate that (for some time) search activity for influenza-like symptoms was an accurate predictor of actual influenza epidemics [37]. A few years later, however, the performance of their algorithms decreased rapidly due to changes in Google’s search algorithms and in the ways people used the search engine [38].

Abebe et al. study health information needs related to HIV/AIDS, malaria and tuberculosis in 54 African countries [39]. Using Bing search data from those countries, the authors show that users are mostly interested in gathering information about symptoms, testing and treatment, but also stigma, discrimination and natural cures. Using 18 months and billions of search queries posted to Bing’s web search engine, Fourney et al. show how concerns about pregnancy and childbirth change over the course of pregnancy [40].

Furthermore, Yahoo! search activity for cancer correlates with estimated cancer incidence, mortality and, especially, news coverage [41]. Similarly, search activity for information about cancer in the U.K. and the U.S. increased from 2008 to 2010, with almost half of all searches dealing with breast cancer, followed by lung and prostate cancer [42]. Most common topics include different treatment forms, diagnosis and screening.

Google Trends, a tool allowing the estimation of aggregated Google search activity for specific queries, is popular for studying health-related search activity [43–45]. For example, research found that queries about breast cancer screening made to Google correlate with changes in legislature and news coverage [44]. Likewise, Google Trends shows that consistent seasonal patterns in search activity exist and that breast, pancreatic and ovarian cancer are among the most searched for forms of cancer [43, 45].

Another approach to obtain information about users’ online search behavior is used in [46]. 56 women were recruited from a commercial vendor in market research. Women answered an online survey and were then instructed to search information online about a hypothetical body change. Monitoring the online searching behavior of the participants through a browser plugin, the authors find that seeking information about unfamiliar symptoms online does not necessarily help women understand their condition.

To sum up, previous work mainly relied on search query data obtained from various search engines or tools built on top of them (Google Trends). One drawback of the latter is that it does not allow analyses on the user level because Google Trends only provides aggregate search activity information. In addition, even if user-level data are available (such as in [36] or [13]), the data does not contain detailed socio-demographic and/or additional health information about users. Moreover, query data is often difficult to obtain [40] or relies on small samples with specific foci [46].

## Data and methodology

We analyze four months (July-October 2017) of web log and mobile device use data for a sample of 1,959 German internet users. Data were collected by a commercial vendor (respondi AG [47] in market research for a third-party (SINUS Markt- und Sozialforschung GmbH [48]) not involved in this study for market research purposes. The vendor keeps an opt-in panel of participants for marketing and social research. Participants of the panel are occasionally invited to answer surveys in exchange for small cash incentives. In addition, participants can agree to the monitoring of their browsing behavior and mobile device use for additional incentives, after giving informed consent that their survey, web log and app use data will be used for academic and market research purposes. Details on the panel, the vendor’s recruitment process as well as the privacy policy can be found at [47].

To ask participants questions, researchers provide them to the vendor who then implements them in its online survey platform. At no times is there contact between researchers and participants of the vendor’s panel. Moreover, the vendor provides all data in pseudonymized and de-identified form. That is, all data accessible to us are striped of users’ names, addresses or birth dates and cannot be linked back.

Given these circumstances (pseudonymized data provided by a survey platform from users of the platform who gave informed consent in combination with no possibility for us to de-identify individuals), this study was exempted from an approval by the Ethics Committee of the University of Mannheim (Ethics Committee of the University of Mannheim, Decision “EK Mannheim 15/2020”).

The vendor gathers web logs from participants’ personal computers and mobile devices (smartphones and tablets) through a tool based on software provided by Wakoopa [49]. Users install a plugin in web browsers used on their personal computers (e.g., Safari, Firefox, Microsoft Edge, Chrome). In addition, they download an app on their mobile devices (smartphones and tablets, Android and iOS devices only). This app collects web logs from the native browsers (i.e., Safari on iOS devices and Chrome on Android devices) as well as information about the apps participants use.

Each time a participant navigates to a website, the complete URL of the website (e.g., https://en.wikipedia.org/wiki/URL), the domain (wikipedia.org), the current date and time as well as the time spent on the website are recorded (both on personal computers and mobile devices). In addition, on mobile devices, information about the apps that participants use are recorded. Every time a participant opens an app on a device, the name of the app, the duration of use and information about the device are logged. Information on activities that individuals perform in an app are not recorded. At any time, participants can turn off data collection temporarily or stop data collection completely.

The vendor also provides background information about the users, including socio-demographics and information on various health issues, which were collected through a web survey. Age, gender, and education quotas were used to achieve a sample approximately representing the German adult population. Table 1 shows characteristics of the participants in our study. Overall, about half of all participants are female (54.72%), and the mean age is about 42 years. 58.96% of participants work full- or half-time. More than 80% have some education beyond basic secondary school and most of the participants have a personal net monthly income between €1,000 and €2,000. Besides socio-demographic information, Table 1 lists also the most common health issues reported by the participants. 32.06%% of all participants indicated back problems and about 26.08% of having (any) allergies. 20.93% reported having high blood pressure, 18.07% problems with sleeplessness, 16.23% depression and 11.84% reported obesity. Regarding other lifestyle behaviors, 41.45% indicated smoking and 76.88% participating in any physical activity (such as running, swimming and playing football). Thus, we find that some participants in the sample do have to cope with several health problems and engage in health-related lifestyle behaviors.

**Table 1:** Descriptive statistics of sample.

*Variable*		*%*
Gender	Female	54.72
	Male	45.28
Age	(mean)	41.87
	(standard deviation)	14.50
Education	Basic secondary school	19.50
	Extensive secondary school	36.35
	High school	23.99
	University degree	20.16
Employment status	Work full-time or part-time	58.96
	Do not work	41.04
Personal income	≤€999	32.01
	€1,000-€1,999	36.86
	≥€2,000	31.14
Health issues	Back problems	32.06
	Allergies	26.08
	High blood pressure	20.93
	Sleeplessness	18.07
	Depression	16.23
	Obesity	11.84
Lifestyle	Smoking	41.45
	Physical Activity	76.88

N = 1,959

Physical activity denotes whether the participant engages with at least one of the following activities: aerobics, badminton, basketball, fitness, football, handball, hockey, jogging, judo, karate, Nordic walking, Pilates, horse riding, swimming, squash, dancing, diving, tennis, volleyball, yoga, cycling or mountain biking, golf, sailing, skiing, surfing.

We create three datasets from the data described above. The first one contains web logs from both personal computers and mobile devices (28,524,036 total logs from 1,959 participants). Overall, participants visited 194,389 unique domains. To identify web logs that refer to domains with health content, we used Webshrinker [50], an online service offering domain categorization. Each of the 194,389 unique domains found in the web logs dataset was categorized into one of the 26 categories of the Interactive Advertising Bureau’s domain taxonomy [51]. If available, Webshrinker also included the appropriate subcategory (for example, chronic pain, dental care or alternative medicine). We then defined a binary indicator denoting whether a domain belongs to the category “health and fitness” or to a different category. Furthermore, we recorded the subcategories for all domains with the “health and fitness” category. Overall, 10,371 out of the 194,518 unique domains (that is, 5.33%) were categorized as being health-related. Table 2 shows the number of health-related domains per subcategory.

**Table 2:** Number of unique domains, by subcategory.

*Subcategory*	*Number of domains*
Abortion	14
AIDS/HIV	30
Allergies	32
Alternative Medicine and Holistic Healing	1,031
Arthritis	12
Asthma	7
Attention Deficit Disorder	2
Autism	25
Bipolar Disorder	6
Brain Tumor	30
Cancer	102
Cholesterol	16
Crohn’s Disease	64
Chronic Fatigue Syndrome	12
Chronic Pain	62
Cold and Flu	48
Deafness	70
Dental Care	423
Depression	25
Dermatology	371
Diabetes	95
Epilepsy	10
Exercise and Weight Loss	1,248
GERD/Acid Reflux	26
Headaches/Migraines	73
Health and Fitness (no subcategory)	3,071
Heart Disease	100
Incest/Abuse Support	113
Incontinence	39
Infertility	29
Men’s Health	396
Nutrition	142
Orthopedics	326
Panic/Anxiety Disorders	30
Pediatrics	93
Physical Therapy	181
Psychology/Psychiatry	700
Senior Health	67
Sleep Disorders	95
Smoking Cessation	53
Substance Abuse	74
Thyroid Disease	50
Vitamins and Food Supplements	576
Women’s Health	402
Total	10,371

Health-related domains only. Web logs dataset.

The second dataset contains records of app use (8,957,760 total records from 1,328 participants). The remaining 631 participants either used a personal computer only or did not use apps on their mobile devices. Therefore, the number of participants in this dataset is smaller than the total number of participants in our study. Overall, participants used 10,123 unique apps. Health-related apps were identified based on the classification of apps used in both Android’s Playstore and Apple’s App Store. All apps classified as “health and fitness” or “medicine” were labelled as health-related. Overall, 476 out of the 10,123 unique apps (that is, 4.70%) were categorized as being health-related. To get a more detailed insight into these apps, we manually coded all apps labelled as health-related into one of the subcategories shown in Table 3.

**Table 3:** Number of apps, by subcategory.

*Subcategory*	*Number of apps*	*Frequently used apps*
Allergies	2	0
Alternative Medicine	3	0
Baby Care	12	4
Beauty Care	1	0
Blood Pressure	2	2
Children	3	0
Dental Care	4	2
Diabetes	9	2
Donate Blood	2	0
First Aid	5	2
General Health Information	31	6
Health Diary Keeping	9	4
Health Insurance	19	4
Health Tracking	69	38
Heart	5	1
Hydration	19	7
Information for Disabled People	1	0
Meditation	27	6
Mental Health	3	0
Migraine	2	1
Neck and Back Problems	2	0
Nutrition	11	2
Palliative Care	1	0
Pregnancy	21	7
Reminder	3	3
Sexual Health	5	2
Sleep	27	5
Smoking Cessation	8	1
Tinnitus	1	0
Unclear	1	0
Veins	1	0
Weight Loss	47	16
Women’s Health	22	13
Workout and Exercise	98	29
*Total*	*476*	*157*

Health-related apps only. Apps dataset. Frequently used apps: Used for at least thirty minutes by at least one user.

Closer inspection of the app use records reveals, however, that only about one third of health apps are actually frequently used (column three of Table 3). Limiting app use records to apps that were used for at least thirty minutes by at least one user (during the four months of data collection), the number of apps decreases to 157. Thus, it seems that many health apps are hardly ever used. Regarding apps that users actually use, we find that tracking one’s health and fitness, as well as exercising and weight loss and women’s health are the popular categories.

The third dataset is a subset of the first one. It contains search queries sent to the 19 most common search engines in the web logs dataset (for example, Google, Bing, Yahoo and DuckDuckGo). From the first dataset, 1,197,421 (4.20%) URLs point to search engines. We extract the search queries from these URLs (for example, the search queries extracted from the URL https://www.google.com/search?q=high+blood+pressure are “high blood pressure”). 1,656 (84.53%) participants used one of the search engines at least once. In order to identify *health*-related search queries in this dataset, we scraped health-related terms from ten German websites. We chose websites that listed diseases and organs and other parts of the human body, technical medical terms, medical disciplines, symptoms of diseases and medical encyclopedia. These websites are listed below. Prior to scraping the terms from the websites, we ensured that our web crawlers were not forbidden on these websites by checking the robots.txt files and the terms and conditions of each domain.

- https://de.wiktionary.org/wiki/Verzeichnis:Deutsch/Medizin
- https://de.wikiquote.org/wiki/Kategorie:K%C3%B6rperteil
- https://de.wiktionary.org/wiki/Verzeichnis:Latein/K%C3%B6rperteile
- https://www.taschenhirn.de/mensch-und-natur/haeufigste-krankheiten-der-welt/
- https://www.taschenhirn.de/mensch-und-natur/organe-des-menschen/
- https://www.netdoktor.de/krankheiten/#Lexikon
- https://www.gelbe-liste.de/krankheiten
- https://flexikon.doccheck.com/de/Liste_von_Medizinprodukten
- https://de.wikipedia.org/wiki/Liste_medizinischer_Fachgebiete
- https://www.onmeda.de/krankheiten/krankheiten_az.html

We then compared string similarity between each search query from the search query dataset and the list of scraped medical terms. We used the fuzzywuzzy Python module for fuzzy string matching (https://github.com/seatgeek/fuzzywuzzy). The module allows the estimation of differences between sequences of characters based on Levenshtein Distances.

Manual inspection of a random sample of matches between the search queries and the list of medical terms revealed that using a sorted token approach with a partial match ratio of 0.7 resulted in a reasonably low number of false positives. That is, we classified those search queries as health-related where the match ratio between a search query and any of the entries from the list of medical terms was at least 0.7. All other search queries were classified as not health-related. With this approach, we classified 9,278 out of 1,197,421 search queries (0.76%) as health-related. 763 users out of the 1,656 users who used a search engine at least ones (that is, 46.07%) searched for health-related information at least once.

## Findings

In this section, we present the main results of our study.

### Health-related internet and app use

1,662 (84.84%) out of 1,959 participants in the web logs dataset visited any health-related domain. Table 4 shows the five most popular subcategories of health-related domains across participants in the sample. Participants seem predominantly interested in information about exercising, losing weight and food supplements, but also in alternatives to traditional medicine. Moreover, mental health and dermatology are popular health topics. A comparison with Table 2 demonstrates that the subcategories with the highest numbers of unique visitors are also among those with the highest number of unique domains.

**Table 4:** Top subcategories of health-related domains and apps.

*Subcategory*	*Number of participants*
**Web Logs Dataset**	**1,959 (100%)**
*Any Health Domain Visited*	1,662 (84.84%)
Exercise and Weight Loss	1,050 (53.60%)
Vitamins and Food Supplements	943 (48.14%)
Alternative Medicine	809 (41.30%)
Psychology/Psychiatry	588 (30.02%)
Dermatology	555 (28.33%)
**Apps Dataset**	**1,328 (100%)**
*Any Health App Used*	494 (37.20%)
Health Tracking	200 (15.06%)
Workout and Exercise	145 (10.92%)
Weight Loss	105 (7.91%)
Women’s Health	100 (7.53%)
*Frequent Health App Use*	224 (16.87%)
Health Tracking	98 (7.38%)
Workout and Exercise	47 (3.54%)
Weight Loss	45 (3.39%)
Women’s Health	20 (1.51%)

Web logs and apps datasets.

494 (37.20%) out of 1,328 participants in the apps dataset used a health app at least once. Using the definition of *frequent* health app use (see Table 3), we find that only 224 (16.87%) out of 1,328 participants participants frequently use health apps. Regarding the most popular subcategories of health apps measured via the number of users, Table 4 shows that users are predominantly interested in monitoring and tracking their health. This finding is likely due to the popularity of wearables that allow, for example, the monitoring of one’s heart rate. Similarly, many participants used an app for exercising and workout, but also apps that focus on weight loss. Furthermore, apps for women’s health are popular. Manual inspection revealed that this subcategory mainly consists of period and ovulation tracker apps. Interestingly, the same categories are also the most popular ones when we restrict the analysis to frequent app users. In addition, the popularity of apps among participants seems to match the popularity of subcategories measured by the number of unique apps in each subcategory (Table 3). Overall, however, more people seem to browse the internet rather than use apps for health-related purposes.

To better understand who uses the internet and apps for health-related purposes, we estimate logistic regression models. Results are shown in Table 5. The models predict a binary variable indicating whether a participant visited any health-related domain, used any health app, used a health app frequently (that is, app use ≥ 30 minutes) or made any health-related search query. Predictors are socio-demographic characteristics, health conditions and lifestyle indicators.

**Table 5:** Odds ratios of logistic regression models predicting health-related online activities.

	*Health domains*	*P value*	*Health apps (freq. use)*	*P value*	*Health apps*	*P value*	*Health searches*	*P value*
Intercept	3.79	<.001	0.71	.27	0.26	<.001	0.50	.01
	(1.98-7.25)		(0.38-1.30)		(0.12-0.60)		(0.31-0.82)	
Age	1.01	.26	0.99	.01	0.98	.02	1.00	.67
	(1.00-1.02)		(0.98-1.00)		(0.97-1.00)		(0.99-1.01)	
Female	1.45	.01	1.17	.20	1.46	.02	1.31	.01
	(1.11-1.89)		(0.92-1.49)		(1.05-2.01)		(1.08-1.60)	
Basic sec. school (*reference*)	—	—	—	—	—	—	—	—
Extensive sec. school	1.11	.57	1.25	.21	1.09	.71	0.88	.35
	(0.78-1.57)		(0.88-1.77)		(0.68-1.75)		(0.68-1.15)	
High school	1.13	.56	1.36	.12	1.35	.24	1.05	.74
	(0.75-1.68)		(0.92-1.99)		(0.82-2.23)		(0.78-1.43)	
University degree	1.35	.17	1.14	.52	0.97	.91	1.53	.01
	(0.88-2.07)		(0.76-1.70)		(0.57-1.66)		(1.13-2.08)	
Employed	0.88	.40	0.95	.70	0.88	.49	1.03	.80
	(0.65-1.19)		(0.71-1.26)		(0.60-1.27)		(0.83-1.28)	
Income: ≤€999 (*reference*)	—	—	—	—	—	—	—	—
Income: €1,000-€1,999	0.98	.90	1.12	.50	1.04	.84	0.93	.57
	(0.68-1.40)		(0.81-1.55)		(0.68-1.59)		(0.72-1.20)	
Income: ≥€2,000	0.91	.63	0.96	.83	1.15	.56	0.83	.21
	(0.62-1.34)		(0.67-1.38)		(0.72-1.82)		(0.63-1.11)	
Back problems	0.95	.77	1.12	.42	0.95	.79	0.90	.34
	(0.70-1.30)		(0.85-1.49)		(0.66-1.38)		(0.72-1.12)	
Allergies	1.24	.17	1.11	.44	1.38	.06	0.94	.58
	(0.91-1.70)		(0.85-1.44)		(0.99-1.91)		(0.76-1.17)	
High blood pressure	1.20	.34	0.85	.35	0.89	.64	1.31	.04
	(0.83-1.73)		(0.60-1.20)		(0.56-1.43)		(1.01-1.70)	
Sleeplessness	1.28	.23	1.21	.30	0.86	.50	1.06	.68
	(0.86-1.90)		(0.89-1.72)		(0.55-1.35)		(0.81-1.39)	
Depression	1.45	.09	1.17	.36	1.29	.26	1.38	.02
	(0.95-2.22)		(0.83-1.65)		(0.83-2.00)		(1.05-1.82)	
Obesity	1.33	.25	1.37	.09	1.30	.27	1.17	.32
	(0.82-2.14)		(0.95-1.99)		(0.81-2.09)		(0.86-1.58)	
Smoking	0.69	.01	0.73	.01	0.54	<.001	0.89	.23
	(0.53-0.89)		(0.58-0.93)		(0.39-0.74)		(0.73-1.08)	
Do any sports	0.88	.40	1.11	.47	1.17	.43	0.97	.80
	(0.64-1.19)		(0.84-1.48)		(0.80-1.71)		(0.78-1.21)	
*Number of participants*	*1,959*		*1,328*		*1,959*			

95% Confidence intervals in parentheses. Frequent use: ≥ 30 minutes use time in total by user.

Columns two and three indicate that women are more likely to browse the internet for health-related purposes, while those who smoke are less likely to seek health information online. Regarding health-related app use (fourth and fifth column), we find that the likelihood of using a health app decreases with age and users who smoke are also less likely to do so. Replacing the dependent variable *any* health app use with *frequent* health app use (columns six and seven), we find similar results. Frequent health app use decreases with age and smoking, but we also find that women are more likely to be frequent health app users. For all three models, we do not find that medical conditions (such as back problems or having a high blood pressure) play a substantial role. The last two columns of Table 5 show results regarding the question who is most likely to *search* for health information using a search engine. Among all internet users in our data, female users are more likely to search health information online as well as users with a university degree. Moreover, those who have reported a high blood pressure and depression are more likely to search information via search engines.

Next, we analyze whether the frequency and duration of using the internet and apps for health-related purposes shows similar patterns. The fine-grained records of online behavior and app use allow us to calculate for each individual how often and how long they used the internet/apps for health-related purposes. We consider the sum of all health-related online activity (that is, the sum of internet use, app use and search engine use). To account for different base levels of online and app activities, we divide each individual’s overall online health activity by the same individual’s overall online and app activity. Table 6 shows the results from the linear regression models. Again, female users spent more of their total online and app activities with health-related activities. Moreover, those individuals who report high blood pressure spent more of their total activity with health-related activities. Similar to the results shown in Table 5, smokers invest less time into health-related online and app activities.

**Table 6:** Linear regression models predicting intensity of health-related online activities.

	*Frequency*	*P* value	*Duration*	*P* value
Intercept	1.512	<.001	1.578	<.001
	(0.436)		(0.477)	
Age	-0.013	.06	-0.013	.08
	(0.007)		(0.007)	
Female	0.536	.002	0.687	<.001
	(0.176)		(0.193)	
Basic sec. school (*reference*)	—	—	—	—
Extensive sec. school	0.067	.78	0.125	.63
	(0.237)		(0.259)	
High school	0.069	.80	-0.167	.57
	(0.272)		(0.297)	
University degree	0.052	.85	0.034	.91
	(0.278)		(0.303)	
Employed	-0.025	.90	0.071	.75
	(0.199)		(0.218)	
Income: ≤€999 (*reference*)	—	—	—	—
€1,000-€1,999	0.001	.99	-0.056	.82
	(0.230)		(0.252)	
≥€2,000	0.125	.62	0.093	.74
	(0.252)		(0.276)	
Back problems	-0.024	.91	-0.157	.48
	(0.202)		(0.221)	
Allergies	-0.025	.90	-0.118	.58
	(0.196)		(0.214)	
High blood pressure	0.542	.02	0.570	.03
	(0.232)		(0.254)	
Sleeplessness	0.092	.71	0.367	.17
	(0.244)		(0.267)	
Depression	0.145	.56	0.207	.45
	(0.250)		(0.274)	
Obesity	-0.073	.79	0.072	.81
	(0.275)		(0.301)	
Smoking	-0.289	.09	-0.385	.04
	(0.172)		(0.189)	
Do any sports	-0.081	.69	-0.002	.99
	(0.200)		(0.219)	
*Number of participants*	*1,959*		*1,959*	

Standard errors in parentheses.

### Health-related search queries

**Fig 1:**
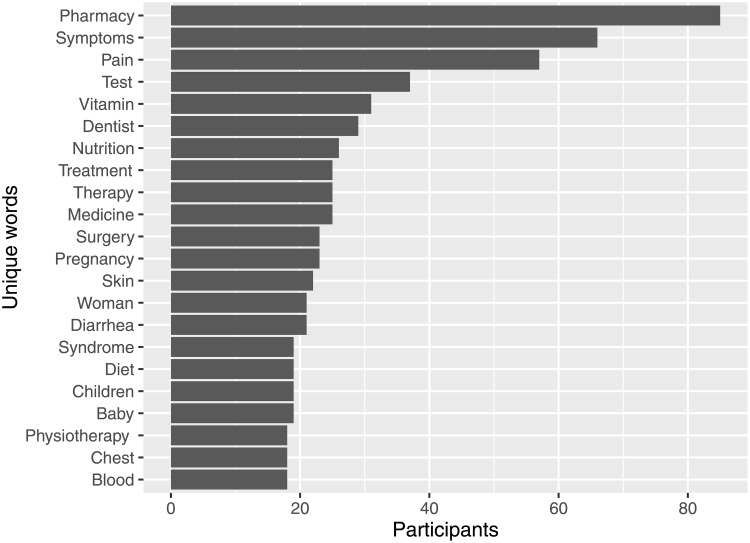


Fig 1 shows the most frequently used words across participants in the search query dataset after removing stopwords. Many participants searched for pharmacies. One motivation may be to find the closest pharmacy, one that is open or to order from an online pharmacy. Moreover, popular terms cover, for example, symptoms, pain and therapy, but also medicine and several organs or body parts (such as, skin and chest). Furthermore, women’s health as well as children and babies were important topics.

In addition to single words, we also considered the most frequently used bigrams (a sequence of two adjacent words) across users (Table 7). Analyzing bigrams allows a better understanding of which topics users search for. Similar to the most frequently used single words, pharmacies are among the most popular bigram words. Moreover, information about specific diseases, but also about rather general health issues (dry skin) and nutrition are sought.

**Table 7:** Most popular bigram words, across users.

*Bigram words*	*Number of participants*
Online pharmacy	38
Ulcerative colitis	8
Crohn’s disease	6
Multiple sclerosis	6
Dry skin	6
Vitamin B12	6
Cataract	5
Ankylosing spondylitis	5
Rheumatoid arthritis	5

Translation by authors. Some German bigrams translate to a different number of words in English.

## Discussion and conclusion

We used a unique combination of records of individuals’ online and app activities with socio-demographic and health information in this study. This dataset provided detailed and fine-grained insights into participants’ internet and app use for health-related purposes. In addition, we observed not only if participants used the internet and/or apps at all, but also to what extent. We studied which health aspects users are most interested in when browsing the internet and using apps. Previous literature had to rely on inaccurate and often incomplete self-reports from surveys. Such self-reports suffer from bias due to users not being able to accurately recall when and how long they used the internet or apps and what exactly they did [35]. Moreover, we also overcame limitations in sample size, while previous research often had to rely on samples limited in size.

Analyzing health-related search queries made to search engines allowed us to study *how* users obtain health information on the internet. Previous studies often had to rely on openly available, but aggregated and less detailed search query data (see, for example, [44]). Obtaining search query data on the user level, for example from search engine providers, is difficult [40] and data, although large in size, do not come with additional information about users’ socio-demographics and health. Obtaining access to data like those used in our study, however, is relatively easy through commercial vendors. While our data and our findings are specific to Germany, similar data are available from providers in many other countries (for example, in the U.S., U.K., Spain and France) which would allow for cross-cultural studies.

### Socio-demographic correlates of internet and app use

Our results on usage patterns across societal groups confirm findings from previous work. First, women are more likely to browse and search for health content online and spend more of their total online activities looking for health information than men. Moreover, women are more likely to be frequent app users. One explanation for these findings may be that women are more often concerned with child rearing than men [52]. That is, women do not only seek information for themselves, but also for their children. Therefore, they spend more time as they have to seek information for more people and people with different needs. Another explanation holds that women are more reactive to deviations from health [17]. Likewise, men tend to wait longer before seeking professional help with their health [53]. Regarding the finding that women are more likely to be frequent health app users, it seems that the popularity of apps for women’s health (such as period trackers, ovulation diaries, and apps concerned with pregnancy) explains this finding. That is, although we found that frequent health app use is rather limited, women seem to profit more than men from technological innovations in the mobile health sector.

Second, we found that younger people and users with a university education are more likely to use health apps and search for health information online. We believe that the effect is driven by better technology literacy among younger and people with a higher education. However, as the likelihood of developing (multiple) chronic health conditions increases with age, older people may actually profit more from using health apps and searching for health information on the internet [54]. Unfortunately, these results do not indicate that internet use helps reducing shortcomings in health knowledge for societal groups with low education [17].

Third, our results show that smoking significantly decreases both the likelihood and the intensity of using the internet and apps for health-related purposes. One explanation for this finding may be that smokers are also more likely to be of lower socio-economic status (that is, lower education and lower income, for example) and more often male [55, 56]. Thus, smokers may be less likely to engage in health-related activities due to the correlation of smoking with other determinants of health-related online activities. However, it is also possible that people who smoke are in general more risk tolerant and thus less concerned with their health [57].

### Contents of internet and app use

The analysis of subcategories of health-related domain reveals that exercising, weight loss and nutrition are among the most popular topics. That is, users seem especially interested in obtaining information about how to live a healthy life. Unsurprisingly, the same categories in addition to health tracking and women’s health are also popular among health apps. These results seem to speak to increasing desires for the ‘quantified-self’, self-optimization through tracking and the analysis of one’s body using health trackers, apps and the like [58].

Another popular topic is mental health. This finding is important as mental health is often associated with stigma and mental health consumers often feel discriminated [59]. Web logs provide new ways of measuring and studying mental health that may be less affected by self-reporting bias (though we note that our measure of depression and sleeplessness rely on self-reported survey data). That is, observing digital traces may offer new means for understanding who may be in need of support and how people may be reached. Moreover, understanding how users gather information may help inform and guide the design of targeted interventions to support those seeking professional help [60]. The popularity of the mental health category also adds to the debate about deficiencies regarding mental health support (such as insufficient availability of psychotherapy) in Germany [61].

Furthermore, we found that alternative medicine is a popular topic among users. This finding seems to confirm reports documenting that Germans are, more than residents from other European countries, particularly susceptible to home remedies [62]. Moreover, it may also supports notions of decreasing trust in evidence-based healthcare and medical experts as expressed through anti-vaccination movements, for example [18, 63]. Against this background, studying how, where and why users turn to alternatives to evidence-based medicine and what content they consume may help develop campaigns aiming to fight the dissemination of inaccurate and potentially dangerous information on the web. Moreover, lay-people often do not have the necessary skills to evaluate the quality of medical information found online. While content may cover professionally or peer-reviewed information, it may also include lay-people’s opinions and anecdotes that might potentially harm users [19, 64]. As mentioned earlier in this paper, the rise of the anti-vaccination movement and similar phenomena is facilitated through information spread through the internet [18]. To study who is susceptible to medical misinformation, future work should, for example, examine the content of medical information consumed by users on the internet. Using detailed records of users’ online activities in combination with scraping and analyzing contents of health websites allows researchers to answer questions that were difficult to study before.

Regarding the analyses of health-related search queries, we note that users often use search engines to seek information about health professionals (such as dentists and physiotherapy) or health institutions (for example, pharmacies). That is, reasons for searching seem primarily functional and once again underline the important role of the internet in facilitating everyday life through the ubiquitous offer of information. In addition, we found treatment-related terms to be among the most popular (such as treatment, therapy and surgery). This finding is somewhat confirmed by the analysis of bigrams, which shows that users often seek information regarding pharmacies, but also about specific diseases. That is, users seem especially interested in obtaining information about specific health issues, but likely also information on treatment options and remedies for conditions they experience. Just as the popularity of health apps, the popularity of treatment-related terms demonstrates users’ growing demand for understanding their body and for pro-actively surveying and managing their own health.

### Limitations

Our analyses of search queries in this paper could only scratch the surface of what we believe is possible with more advanced natural language processing algorithms. However, we also note that the automated extraction, categorization and understanding of health-related search query data is challenging, especially when it comes to inferring user intentions. For example, a person searching for “ulcerative colitis” may seek information about the disease because she has never heard of it, she may worry that she has the disease due to specific symptoms she experiences or she may have the disease and seek, for example, information on treatments. Understanding which intentions users have when searching for health-related information may, for example, require studying whole browsing sessions. In addition, studying developments of anti-vaccination positions, for example, will likely require the observation of even longer time periods. Moreover, analyzing specific health topics may also require observing larger samples as the number of observations will rapidly decrease if those topics are not widespread in the population.

### Future work

Besides substantive interest in health research, we believe that studying health-related internet and app will prove important from a privacy perspective. Privacy research documents that website tracking and sharing of sensitive personal information, which includes health information, is a widespread phenomenon in the online and app world [65–69]. For example, more than 76% of popular web pages that offer information and support regarding mental health contain third-party tracking elements (such as cookies) for marketing purposes [66]. Thus, sensitive personal information on users’ mental health may be observable for third parties. Yet, information about such tracking practices provided to users of web pages or apps often does not meet the privacy regulations required under the European Union’s General Data Protection Regulation or under the ePrivacy law. Some web pages and apps even fall short collecting users’ informed consent at all when sharing personal sensitive information with third parties [65–67]. In addition, users often do not understand what information is collected about them when browsing websites or using health apps. Even if privacy policies are provided, users rarely read them [70]. It is therefore crucial to understand whether third parties may be able to infer users sensitive information like their health status by observing, for example, her online activities through cookies and tracking in apps and on mobile devices [69].

In light of the current COVID-19 pandemic, it seems more important than ever to study where and how people search for health information, what kinds of websites they visit or apps they use, and critically examine the quality of information users find given that plenty of (mis)information is disseminated online. Passively tracked browser and app use data, such as the data used in the present study, might be a promising way to shed more light into this.
